# The Deteriorated Center-Surround Suppression in Patients With First-Episode and Drug-Naïve Major Depressive Disorder

**DOI:** 10.1155/da/7376934

**Published:** 2025-05-20

**Authors:** Yunyue Zhuang, Weijie Song, Shenbing Kuang, Wei Li, Shujuan Pan, Zhiren Wang, Wei Qu, Jingxu Chen, Yunlong Tan, Chundi Wang, Hu Deng

**Affiliations:** ^1^Department of Otolaryngology, Head and Neck Surgery, National Center for Children's Health, Beijing Children's Hospital, Capital Medical University, Beijing, China; ^2^Peking University Huilongguan Clinical Medical School, Beijing Huilongguan Hospital, Beijing, China; ^3^State Key Laboratory of Cognitive Science and Mental Health, Institute of Psychology, Chinese Academy of Sciences, Beijing, China; ^4^Department of Psychology, School of Humanities and Social Sciences, Beihang University, Beijing, China

**Keywords:** center-surround suppression, deterioration, first-episode and drug-naïve, major depressive disorder, motion repulsion

## Abstract

**Background:** Major depressive disorder (MDD) patients are often associated with inhibition deficits in the visual cortex. Most previous research has focused on visual inhibition in MDD patients during acute and remission phases, with little research on first-episode and drug-naïve (FEDN) patients. To fill this gap, we psychophysically investigated the inhibitory process of visual motion in patients with FEDN MDD.

**Methods:** Two psychophysical tasks: Center-Surround Suppression (CSS) and Motion Repulsion (MR) were utilized to investigate the presence of visual perceptual inhibition deficits in patients with FEDN MDD. We collected data from 74 patients with FEDN MDD and 68 healthy controls (HCs) matched for age, years of education, and gender. We also measured the Hamilton Depression Rating Scale, 17-item (HAMD-17) for each patient to assess the severity of depressive symptoms.

**Results:** The results showed that CSS was significantly deteriorated in FEDN MDD patients (*p*=0.001), but it did not correlate with the HAMD score (*p* > 0.05). In addition, no significant differences were observed between the two groups of subjects in terms of gender, age, and education level (all *p* > 0.05). Analysis of two previously published datasets using the same CSS task involving MDD patients in different illness stages revealed that the levels of CSS reduction in our FEDN patients were also significantly distinguishable from those in acute and recovered MDD patients (all *p* < 0.01). This quantitative comparison indicates that CSS impairments are dynamic over the course of illness development. Moreover, the magnitudes of MR showed no significant differences between MDD patients and HCs (all *p* > 0.05).

**Conclusions:** Our study was the first to demonstrate the deteriorated CSS in patients with FEDN MDD. Notably, inhibitory deficits in MDD are also highly specific, as MDD affects only the CSS, but not the MR. Therefore, the discrepancy between these two psychophysical tasks suggests CSS may serve as a feasible early marker in MDD. These findings offer new insights into the specific visual cortical deficits in patients with FEDN MDD.

**Limitation:** The current study lacks imaging data to support the perceptual phenomenon we observed.

## 1. Introduction

Major depressive disorder (MDD) is the most common and globally prevalent mental illness. According to the World Health Organization, MDD will become the leading global burden of disease by the year 2030 [1]. In China, depressive disorders were more common among women than men, and ~20.3% of the older adults receiving primary care met the Diagnostic and Statistical Manual of Mental Disorders (DSM)-IV criteria for depressive disorders [2, 3]. In particular, the COVID-19 pandemic is estimated to result in an additional 53.2 million (44.8–62.9) cases of MDD worldwide, reflecting a 27.6% (25.1–30.3) increase [4]. MDD is typically characterized by persistent depressed mood, loss of interest in pleasurable activities, sleep disorders, and impairments of perceptual and cognitive functions across different domains [3, 5, 6]. Although there has been considerable research progress in understanding visual perceptual deficits in MDD [7–10], whether these impairments are present in patients with first-episode and drug-naïve (FEDN) MDD remains unclear. Identifying these impairments could be an important avenue for earlier intervention and enhancing the overall prognosis of patients with FEDN MDD.

Cognitive impairment is an important feature of individuals with MDD. The meta-analysis by Zhong et al. [10] demonstrated that, compared to healthy controls (HCs), patients with unipolar depressive disorder exhibited significantly longer P300 latency and lower amplitude, suggesting potential deficits in cognitive information processing. Previous studies have reported the persistence of functional and cognitive deficits (in the domains of visual inhibition, visual attention, and working memory) in individuals with recurrent depression [11–13]. At the perceptual level, several studies have utilized physiological and behavioral measures to investigate visual inhibition in patients with MDD. These studies have demonstrated that center-surround suppression (CSS), a well-known perceptual phenomenon of visual inhibition, is significantly impaired in patients with MDD [8, 9, 14]. However, Norton et al. [15] found that depressed individuals exhibited significantly greater CSS effects compared to HCs. These inconsistencies in previous findings may be due to the fact that the depressive populations studied were at different stages of the illness, combined with the effects of medication [16]. Therefore, the controversy of these findings regarding the visual perceptual inhibition has necessitated further investigation in patients with FEDN MDD, who are less influenced by medications and the course of the illness, especially the long-term medication effects.

Also, increasing evidences suggest that the visual cortex plays a crucial role in the association between visual inhibition function and depression [7, 9]. And two perceptual phenomena are linked to the inhibitory mechanisms of the visual cortex. One is the CSS, it is defined as a higher threshold for an individual to detect a large high-contrast Gabor compared to a small high-contrast Gabor [17]. Patients with depression showed CSS deficits and reductions in *γ*-aminobutyric acid (GABA) levels in the occipital visual cortex such as the middle temporal visual area (MT/V5) [9, 18–21]. However, the dynamic characteristics of CSS deficits across different stages of illness should be warranted.

Alongside the study of CSS, the other compelling perceptual phenomenon that demonstrates inhibitory functions in visual motion processing is motion repulsion (MR) [22, 23]. MR is the phenomenon in which observers overestimate the physical angle relative to the actual angle when two motion stimuli are acutely oriented [24–26]. This phenomenon of the greater perceptual than physical orientation is attributed to close mutual inhibition between neurons regulating adjacent orientations within the same cortex [25–28]. The impairment of CSS has been the main focus of previous studies of inhibitory function in MDD patients. So far, it remains unclear whether MDD affects all types of inhibitory functions in the brain, or only causes CSS-specific inhibition dysfunctions [14, 15, 22]. Therefore, this study employed the CSS and MR to demonstrate the pure effects of MDD on the distinct visual inhibitory functions in patients with FEDN MDD.

It has been repeatedly reported that there is a decreased visual inhibition function in patients with MDD. Nevertheless, the effect of medication cannot be ruled out [8, 9]. To our best knowledge, few study has yet investigated the profiles of CSS and MR in patients with FEDN MDD. The study of patients with FEDN MDD offers a unique advantage in understanding changes in visual inhibition functions caused by the illness, as it may help to minimize confounding factors such as long-term medication, duration of illness effects, and comorbidities associated with chronic MDD. We hypothesized that: (1) CSS would be reduced in patients with FEDN MDD compared to HCs; (2) CSS and MR would exhibit different trends in patients with FEDN MDD.

## 2. Materials and Methods

### 2.1. Participants

The interpretation of studies on MDD patients could be complicated by multiple factors, such as the effect of treatments, alterations in treatment measures, or other variables. To eliminate the influence of these factors, we carefully selected a set of patients with FEDN MDD and paired them with HCs. The study included 74 adult individuals with FEDN MDD and 68 adult HCs. We matched the recruited patients with FEDN MDD and HCs based on gender, age, and years of education.

The inclusion criteria of FEDN MDD patients were as follows: (1) diagnosis of the psychiatrists according to the DSM-(V) Fifth Edition [29]; (2) Hamilton Depression Rating Scale, 17-item (HAMD-17) score >7; (3) age range: 18 years–30 years; (4) normal or corrected to normal vision; (5) first episode of depression and no previous treatment with antidepressant medication. The exclusion criteria of FEDN MDD patients were as follows: (1) diagnosis of other psychiatric disorders; (2) vision disorders such as color blindness and color weakness; (3) severe neurological disorders or intellectual retardation; (4) drug/alcohol abuse or addiction; (5) Serious physical illness. The demographics and clinical characteristics of the MDD group can be seen in Table 1.

All experimental protocols were approved by the Human Ethics Committee of Beijing Huilongguan Hospital and conducted in accordance with institutional ethical guidelines. Written informed consent was obtained from all subjects.

### 2.2. Measures

#### 2.2.1. Clinical Measures

HAMD-17: The HAMD-17 is a scale that measures 17 dimensions of depressive symptoms. The scale consists of 17 items, each rated on a scale of 0–2 or 0–4. The higher the score, the more serious the indication [30]. This scale is an excellent tool to test the severity of depression, which has demonstrated robust reliability and validity of the scale in the Chinese population [31].

#### 2.2.2. Procedures of Experimental Paradigms

##### 2.2.2.1. CSS

**Figure 1:**
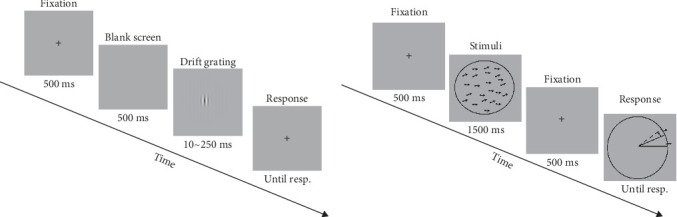
Two different visual inhibition experimental paradigms (A) the CSS paradigm. A fixation point was first started, followed by a blank screen for 500 ms. The presentation duration of the sinusoidal Gabor was then based on a 3-up 1-down staircase procedure. After the stimulus disappeared, a fixation point appeared on the screen. Participants were required to report the direction of Gabor drift by pressing one of two response keys. (B) the MR paradigm. Each trial started with a 500 ms fixation point accompanied through two superimposed moving RDKs. The reference RDKs moved horizontally to the right. Participant were required to report the perceived moving direction of the target RDKs. Until resp. represents the until response.

In Experiment 1, a drifting Gabor was used as the visual stimulus to assess the inter-cortical inhibition. The visual stimuli and experimental procedures were similar to those in the previous studies [17, 23]. The visual stimuli were presented on a Dell computer with a screen resolution of 1920 × 1080 and a refresh rate of 120 Hz. Subjects were seated 47 cm from the screen and answered via an external keyboard. Stimulus contrast was modulated by a two-dimensional Gaussian envelope, either 5% or 95%. The visual stimuli subtended 1°, 2°, 4° of visual angle at the viewing distance. A total of six blocks were included, depending on the combination of contrast and stimulus size. The order in which the six blocks were presented was randomly balanced. We set up two interleaved staircase procedures in every block, each of which converged to the duration required to produce a 79.4% correct rate. The total number of trials was set at 150. The maximum luminance of the visual stimuli was 29 cd/m^2^ (Figure 1A).

##### 2.2.2.2. MR:

In Experiment 2, the random dot kinematograms (RDKs) were used as the visual stimuli in the MR task to assess the repulsion effect. The visual stimuli were presented on a Dell computer with a screen resolution of 1920*⁣*^*∗*^1080 and a refresh rate of 60 Hz. The black dots in the visual stimuli had a diameter of 0.1° and a coherence of 100%, moving against a circular background. The RDKs moved at a speed of 3°/s, and the diameter of the circular background was 4.9°. The reference RDKs were kept moving horizontally to the right throughout the experiment, while the target RDKs moved in varying directions (the actual angles between two motion directions were 5.6°, 22.5°, 45°, 67.5°, 90°, 135°, and 180°). Participants were asked to report the perceived motion direction of target RDKs by clicking on the external mouse. There were 16 blocks in experiment 2 and the order of stimuli presentation was randomized (Figure 1B). The visual stimuli and experimental control stimuli in Experiment 1 and 2 were generated in MATLAB using the Psychophysics Toolbox extension [32–34].

#### 2.2.3. Control of Confounding Factors

To minimize environmental interference, the experiments were carried out in a controlled laboratory environment that was dark, quiet, and separate. Participants were required to complete the task individually to avoid distractions.

Prior to the initiation of the experimental procedure, the participants were allotted a sufficient amount of time to thoroughly understand the instructions of the two experiments. Concurrently, they were required to engage in the nonexperimental condition for practice, which preceded the formal experiment, until they attained complete comprehension of the experimental tasks and the practice trials were excluded from the data analysis.

Since the fact that the total duration of the two experiments was 40–60 min, it was challenging for the participants to complete the entire experiment at one time. Consequently, two experiments were segmented into 2–4 parts to complete. Multiple mandatory rest periods were set, which intended to avoid the potential fatigue effects.

### 2.3. Statistical Analysis

In Experiment 1, the duration threshold was calculated as the mean of the final 6 reversal points, and the thresholds from the two staircases were averaged. The suppression index (SI) was calculated as log_10_ (large threshold) −log_10_ (1° threshold). The difference in the SI and discrimination threshold between HCs and MDD was assessed by a two-way mixed design analysis of variance (ANOVA). Stimulus size (1°, 2°, 4°) served as a within-subjects factor, and group (HCs, MDD) served as a between-subjects factor.

In Experiment 2, we used a two-way mixed-design ANOVA to compare the magnitude of the MR between HCs and the MDD group. The magnitude of the MR was defined as the angular difference between the perceived and physical directions.

Significance of all statistical analyses was determined using a two-tailed test with *p* < 0.05. All statistical analyses for this study were performed using Statistical Package for the Social Sciences (SPSS) 26.0.

## 3. Results

### 3.1. The Deteriorated CSS in Patients With FEDN MDD

**Figure 2:**
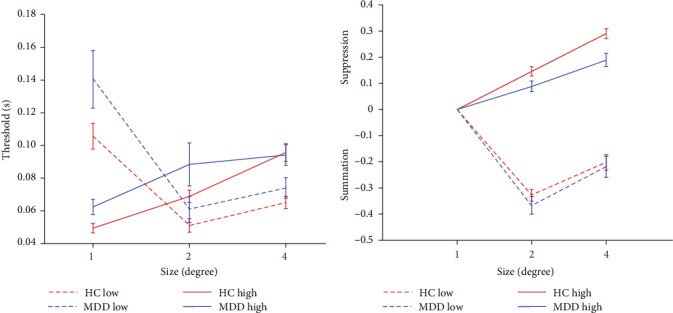
(A) the discrimination threshold for both groups of participants. Red indicates the HCs and blue indicates the MDD group. The dashed line represents 5% contrast and the solid line represents 95% contrast. The error bar represents the standard error of the mean. (B) the surround suppression index for both groups of participants. Red indicates the HCs and blue indicates the MDD group. The dashed line represents 5% contrast and the solid line represents 95% contrast. The error bar represents the standard error of the mean.

To examine the effects of depression, we utilized the CSS task, which was derived from the experimental paradigm previously established by Deng et al. [23] and Betts et al. [35]. All the participants were asked to complete 6 blocks of experiments under different conditions and to finally calculate their discrimination thresholds. The lower threshold represents a higher ability to discriminate the direction of the drifting Gabor. At 95% contrast, an ANOVA analysis of the thresholds revealed a significant main effect for size (*F* = 17.24, *p* < 0.001, *η*_*p*_^2^ = 0.077), but the main effect for the group was not significant (*F* = 3.528, *p*=0.061, *η*_*p*_^2^ = 0.008). In addition, the interaction between group and size was not significant (*F* = 1.292, *p*=0.276, *η*_*p*_^2^ = 0.006). As shown in Figure 2A, when the stimulus size was 2°, there was a significant difference for the discrimination threshold between the two groups (*p*=0.044, Bonferroni corrected). These results suggested that patients with FEDN MDD tended to have a reduced ability to discriminate the direction of large motion stimuli (stimulus size: 2°).

Increasing the diameter of the stimulus size makes the direction of the drifting high-contrast Gabor significantly more difficult to perceive and raises the discrimination threshold, a perceptual phenomenon known as ‘spatial suppression' [17, 36]. The SI is calculated as log_10_ (large threshold) − log_10_ (1° threshold).

As shown in Figure 2B, further analysis of the SI revealed that the both main effect of the group (*F* = 14.355, *p* < 0.001, *η*_*p*_^2^ = 0.05) and main effect of the stimulus size (*F* = 34.152, *p* < 0.001, *η*_*p*_^2^ = 0.112) were significantly associated with the main effect of stimulus size in the high contrast condition. Further pairwise comparison analysis revealed that the SI of 4° stimulus size in patients with FEDN MDD (mean: 0.189; standard deviation (SD): 0.198) was significantly lower than that in HCs (mean: 0.290; SD: 0.163; *p*=0.001, Bonferroni corrected). These results suggested that patients with FEDN MDD had impaired CSS compared to HCs.

### 3.2. The Correlation Between CSS Index and Depressive Symptoms and Comparison of CSS Index Across Different Periods of MDD

**Figure 3:**
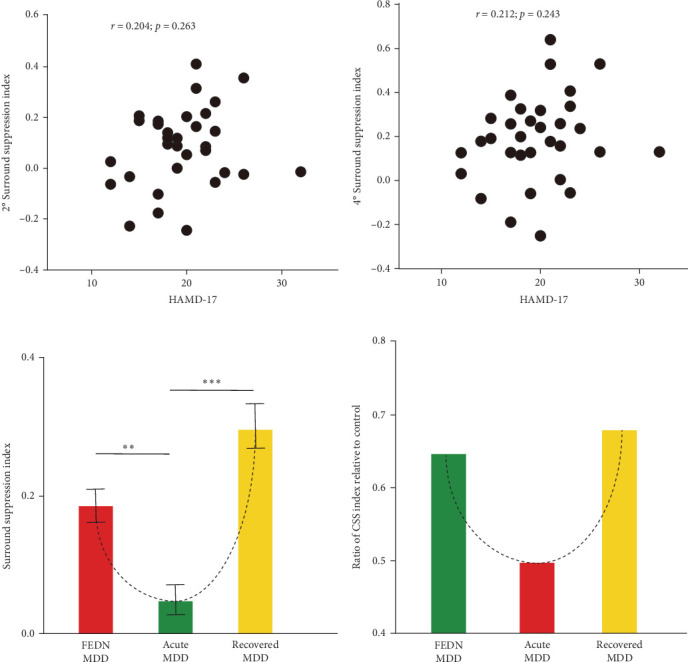
(A) relationship between the surround suppression index of 2° Gabor stimulus and score of HAMD-17 in patients with FEDN MDD. (B) relationship between the surround suppression index of 4° Gabor stimulus and score of HAMD-17 in patients with FEDN MDD. (C) the comparison of surround suppression index among different periods of MDD for high contrast condition. Acute MDD data were replotted from Song et al. [9] and recovered MDD data were replotted from Golomb et al. [8], respectively. (D) the comparison of the ratio of CSS index relative to health controls from our, Song et al. [9]and Golomb et al. [8] studies among different periods of MDD for high contrast condition. The error bar represents the standard error of the mean. *⁣*^*∗∗*^ indicates *p*-value <0.01, and *⁣*^*∗∗∗*^ indicates *p*-value <0.001.

To understand the relationship between the severity of depressive symptoms and the strength of surround suppression in FEDN MDD patients, we performed correlational analyses for 2° stimulus size and 4° stimulus size. Unfortunately, neither 2° stimulus size (Figure 3A) nor 4° stimulus size (Figure 3B) varied with the high-contrast surround SI (*r* = 0.054; *p*=0.682, and *r* = 0.097; *p*=0.448, respectively). These results suggested that there was a congruent impairment of surround suppression in patients with FEDN MDD, independent of the severity of MDD at the early stage.

Therefore, we further explored the dynamic characteristics of surround suppression across different disease cycles (first episode, acute episode and remission). This analysis was performed with the data from this study and two previous studies [8, 9]. The number of participants, mean values, and standard deviations of surround SI were derived from two previous studies. There were 16 subjects with recovered MDD (mean = 0.3, SD = 0.13) and 17 subjects with acute MDD (mean = 0.05, SD = 0.09). Summary *t*-test with Bonferroni correction was conducted using the mean value and the standard error of the mean. This analysis showed that the CSS index during the acute episode exhibited a significantly sharp decrease when compared with the first episode and remission (all *p* < 0.05). The CSS index showed a downward trend at first, and then an upward trend over the entire course of the disease (Figure 3C). To exclude the influence of different visual stimulus parameters adopted by these studies, we also calculated the ratio of CSS index in MDD relative to HCs. We also found a similar trend in the ratio of CSS index (Figure 3D). Taken together, these results suggested that the effect of MDD on surround suppression varied depending on the course of the illness.

### 3.3. The Absence of MDD Impact on MR

**Figure 4:**
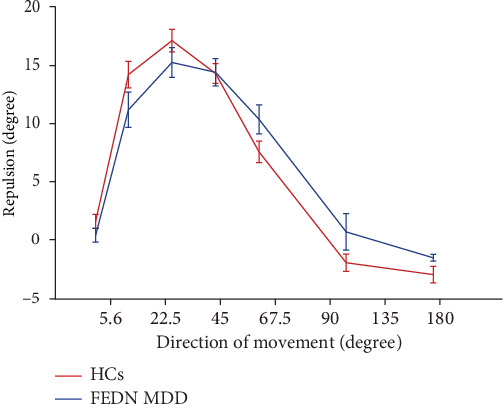
The motion repulsion effect for both groups of participants. The motion repulsion was calculated as the target RDKs for the difference between the perceived and actual angle. Red indicates the HCs and blue indicates the MDD group. The error bar represents the standard error of the mean.

We also utilized the MR task to investigate the effects of FEDN MDD on visual perceptual inhibition. As shown in Figure 4, the magnitude of the MR increased and then decreased for both groups of participants as the direction of movement varied from 5.6° to 180°. In detail, significant MR effects were observed in the HCs at 5.6°, 22.5°, 45°, 67.5°, and 90° (greater than 0) (*t* = 2.721, 13.686, 19.131, 18.312, 8.478, −2.577, −4.738; all *p* < 0.05) and 135° and 180° (less than 0) (*t* = −2.577, −4.738). For the MDD group, significant MR effects were observed at 22.5°, 45°, 67.5°, 90° (greater than 0) (*t* = 8.61, 13.057, 12.736, 8.277; all *p* < 0.001) and 180° (less than 0) (*t* = −4.702; *p* < 0.001), but not at 5.6° and 135° (*t* = 1.336, 0.504; all *p* > 0.05).

We did not find a significant main effect of group (*F* = 0.035, *p*=0.852, *η*_*p*_^2^ < 0.001). Further pairwise comparison revealed that there was no significant difference between the FEDN MDD group and the healthy control group for each direction of movement (all *p* > 0.05). This is inconsistent with our research hypothesis. These results showed no significant difference in the MR effect between the patients with FEDN MDD and HCs.

## 4. Discussion

In this study, we investigated for the first time the effect of MDD on CSS and MR in patients with FEDN MDD. This allowed us to elucidate the effect of pure illness factors on visual inhibitory function, by minimizing the influences of medication, disease course and comorbidities on these outcomes. By adopting two visual inhibition tasks, our main findings are as follows: (1) the perceptual visual inhibition, CSS, was significantly impaired in the patients with FEDN MDD. (2) surround suppression did not correlate with the severity of depressive symptoms during the first episode of MDD, but may fluctuate dramatically over the entire course of illness. (3) regarding the other visual perceptual inhibition, MR, showed no significant difference between patients with FEDN MDD and HCs.

Using two psychophysical tasks, our study provided the first evidence that the function of the visual cortex in the patients with FEDN MDD, especially the CSS, is impaired. This is consistent with our hypothesis. Previous studies had also confirmed a decrease in SI in patients with recovered MDD and those with acute MDD taking medication [8, 9, 21]. Compared with the previous studies by Song et al. [9] and Liu et al. [21], our study here provides a simpler, more direct, and medication-free way to measure the deterioration of visual inhibition in patients with MDD. More importantly, considering the study of Golomb et al., we also provide a brief profile of the visual inhibition function in the patients with first-episode MDD. These findings could help us recognize, identify, and intervene in cognitive deficits in MDD from the perspective of the overall course of the illness.

Although another study [15] stands in contrast to our findings and previous studies mentioned above, and the visual cortex has not traditionally been considered a key contributor to the neural circuitry of MDD, we still provide the support for the role of visual cortex as a candidate biomarker. There are two possible explanations for this phenomenon. First, the inhibition of feedback from higher cortices to lower inter-cortical brain circuits might be impaired in MDD patients. Some researchers have suggested that CSS is a ubiquitous property of visual cortex that is associated with the inhibition of the low-level cortical areas by high-level cortical areas [22, 37]. Previous studies have found a significant reduction in visual inhibition following the inactivation of the V5 in animals [38–40]. Moreover, Tadin et al. [41] further identified this mechanism of surround suppression in human subjects using transcranial magnetic stimulation. Most importantly, a recent study provided strong evidence supporting this speculation. Hu et al. [42] found a significant reduction in functional connectivity between the high-level cortical areas – MT/V5 and other visual areas in patients with FEDN MDD, which was also associated with inhibition control. Therefore, according to the theory of synaptic weakening in MDD [43], the impaired feedback inhibition from higher cortical to lower inter-cortical brain circuits in FEDN MDD may contribute to the deterioration of surround suppression.

Second, the low level of GABA in the occipital cortex and the imbalance between GABA and glutamic acid (GLU) in MDD may provide another explanation. Reductions in inhibitory GABA levels and the GABA-GLU imbalance have been identified in vision-related cortices in MDD patients, particularly in the MT/V5 area [9, 20, 44, 45]. The reduction in GABA levels in FEDN MDD [46, 47], leading to a disruption of GABA-GLU balance (excitation-inhibition balance) [47] and the inactivation of inhibitory neurons [48], may contribute to the impairment of inhibitory feedback connections from MT/V5 area to low-level visual area [49]. Based on our findings and previous mechanistic studies, it is possible that the diminished CSS in patients with FEDN MDD may be related to decreases in GABA levels and disruptions in feedback inhibition of brain circuits between higher and lower cortical levels.

In our analysis, it should be noted that the CSS index showed a downward trend in the acute phase and then an upward trend in the remission phase, when compared to the FEDN MDD. Despite the lack of direct measurements of GABA concentrations across these stages, this is an interesting phenomenon, particularly the downward trend in the acute phase. Patients with first episode, unmedicated MDD exhibit the significantly lower GABA levels in occipital and ventromedial prefrontal cortices [46, 47]. Nevertheless, in the acute phase of MDD, the findings are mixed and elusive. A meta-analysis [50] and previous studies [9, 19, 51] have reported significantly lower GABA levels in currently depressed patients compared to the control group. However, considering the effects of medication, selective serotonin reuptake inhibitors (SSRIs) in MDD have been shown to increase GABA levels in occipital cortex [52]. Hence, in the acute phase, the challenge of ruling out the effects of medication on GABA concentrations still remains. In studies measuring GABA levels in remitted MDD, no significant difference was observed between MDD patients and controls [50]. This suggests that there is a normalization of GABA concentration when patients achieve remission depressive symptoms. Notably, these aforementioned studies measuring GABA concentrations in patients with MDD did not establish a link between visual perceptual inhibition and GABA concentrations. Our interpretations with respect to GABA are based on previous literature and should be regarded as speculative in the absence of direct measurements. Further larger-scale studies comparing the GABA concentration across the three different clinical states above should be warranted.

In contrast to the deteriorated surround suppression in FEDN MDD, we found that the MR effect was not affected in patients with FEDN MDD. This is the first study to investigate the MR effect in MDD patients. The MR effect reflects mutual inhibition between neighboring neurons tuned for similar motion directions within motion sensitive cortical areas [25, 27, 53]. Regarding the MDD serving as an independent risk factor for developing Alzheimer's Disease [54], plus the weakened MR effect in Alzheimer's Disease patients [55], we hypothesized that the deteriorated MR effect could be observed in MDD. However, our study found no significant differences in the MR effect between patients with FEDN MDD and HCs. These results might suggest that, in terms of MR, the inhibitory processing of patients with FEDN MDD did not differ significantly from that of HCs.

## 5. Limitations

Several limitations warrant consideration. First, although two psychophysical tasks were used to measure the visual inhibition in patients with FEDN MDD, our study lacks magnetic resonance imaging (MRI) evidence in our study to support the potential mechanisms proposed in the Discussion section. Future studies should combine behavioral tasks with imaging methods to address this limitation. Second, concerning it is challenging to dismiss the likelihood that the patient may eventually develop bipolar disorder within the foreseeable future, the implementation of a longitudinal follow-up study could provide more robust support for these findings of this study. Additionally, further studies should directly examine surround suppression using consistent visual stimulus parameters and compare GABA concentrations across different stages within a single study. Although the current study compared and discussed them, our elaboration should be cautious and must be considered preliminary. Future studies could be conducted longitudinally, examining the onset and prognosis of MDD to investigate changes in inhibitory function. Third, while the experimental tasks used in this study were feasible in a controlled research setting, their implementation in clinical practice may be challenging due to the complexity of the procedures. Future studies should aim to develop more practical and accessible versions of these tasks to enhance their feasibility in real-world clinical applications.

## 6. Conclusions

In general, the present study reveals that patients with FEDN MDD exhibit the deteriorated surround suppression. In addition, no correlation was observed between the weakened CSS and the severity of depressive symptoms in FEDN MDD, but the impaired CSS may fluctuate dramatically over the entire course of illness. In contrast to the deterioration of surround suppression, the MR effect in patients with FEDN MDD is similar to those of the HCs. Therefore, our findings have important implications for clinical practice, as the identification and treatment of visual inhibition in early acute phase of MDD may lead to better outcomes. The surround suppression may serve as a potential neuropsychological biomarker of FEDN MDD.

## Figures and Tables

**Table 1:** Clinical information of the samples.

Characteristic	MDD (*n* = 74)	HCs (*n* = 68)	*p*-Value
Gender (M/F)	33/41	24/44	0.259
Age, years (SD)	24.18 (6.432)	23.46 (2.028)	0.382
Education, years (SD)	17.53 (2.836)	17.84 (1.98)	0.451
HAMD-17 scores (SD)	17.49 (5.039)	N/A	—
Course of the disease (month, SD)	18.06 (26.456)	N/A	—

*Note:* The data are represented as Mean (SD).

Abbreviations: HAMD-17, Hamilton Depression Rating Scale, 17-item; HCs, healthy controls; MDD, Major Depressive Disorder.

## Data Availability

The data that support the findings of this study are available from the corresponding author upon reasonable request.
